# Antikinetoplastid Activity of Sesquiterpenes Isolated from the Zoanthid *Palythoa aff. clavata*

**DOI:** 10.3390/ph14111095

**Published:** 2021-10-28

**Authors:** Carlos J. Bethencourt-Estrella, Nathalia Nocchi, Atteneri López-Arencibia, Desirée San Nicolás-Hernández, María L. Souto, Blanca Suárez-Gómez, Ana R. Díaz-Marrero, José J. Fernández, Jacob Lorenzo-Morales, José E. Piñero

**Affiliations:** 1 Instituto Universitario de Enfermedades Tropicales y Salud Pública de Canarias (IUETSPC), Universidad de La Laguna (ULL), Avenida Astrofísico Francisco Sánchez s/n, 38203 La Laguna, Spain; cbethene@ull.edu.es (C.J.B.-E.); atlopez@ull.edu.es (A.L.-A.); dsannico@ull.edu.es (D.S.N.-H.); 2 Departamento de Obstetricia y Ginecología, Pediatría, Medicina Preventiva y Salud Pública, Toxicología, Medicina Legal y Forense y Parasitología, Universidad de La Laguna (ULL), Avenida Astrofísico Francisco Sánchez s/n, 38203 La Laguna, Spain; 3 Red de Investigación Cooperativa en Enfermedades Tropicales (RICET), 28029 Madrid, Spain; 4 Instituto Universitario de Bio-Orgánica Antonio González (IUBO AG), Universidad de La Laguna (ULL), Avenida Astrofísico Francisco Sánchez 2, 38206 La Laguna, Spain; nathalianocchi@ull.edu.es (N.N.); msouto@ull.edu.es (M.L.S.); blanca.suarez@gmail.com (B.S.-G.); 5 Departamento de Química Orgánica, Universidad de La Laguna (ULL), Avenida Astrofísico Francisco Sánchez s/n, 38203 La Laguna, Spain; 6 Consorcio Centro de Investigacion Biomedica en Red M.P. (CIBER) de Enfermedades Infecciosas, Instituto de Salud Carlos III, 28029 Madrid, Spain

**Keywords:** kinetoplastids, *Trypanosoma*, *Leishmania*, leishmanicidal, Chagas, trypanocidal, *Palythoa*, sesquiterpenes, chemotherapy, programmed cell death

## Abstract

Leishmaniasis and Chagas disease are neglected tropical diseases that cause problems in developing countries. The causative agents, *Leishmania* spp. and *Trypanosoma cruzi*, produce a clinical picture that can be fatal for the patient, such as Chagas heart disease, visceral leishmaniasis and megacolon, among others. Current treatments for these diseases are not very effective and highly toxic, since they require very prolonged treatments. The development of innovative, effective and safe drugs to fight infections caused by these parasites remains a challenge. For this reason, in recent years, there has been an increase in the search for new therapies. In this study, the antikinetoplastid activity of 13 sesquiterpene lactones obtained from *Palythoa aff. clavata* was screened against *L. amazonensis*, *L. donovani* and *T. cruzi*. The results revealed that the sesquiterpene lactones anhydroartemorin (**2**), *cis,trans*-costunolide-14-acetate (**3**) and 4-hydroxyarbusculin A (**11**) were the most selective against the kinetoplastid species studied. These molecules seem to induce the mechanisms involved in an apoptotic-like death or programmed cell death (PCD) in the kinetoplastids, and since they do not cause necrosis, the inflammatory events associated with this type of cell death will not be triggered.

## 1. Introduction

Leishmaniasis and Chagas disease, whose causative agents are *Leishmania* spp. and *Trypanosoma cruzi*, respectively, are recognized as neglected tropical diseases by the World Health Organization (WHO), partly due to the lack of support and investment of the pharmaceutical industry and the low public visibility they have in developed countries [1,2,3]. The currently available treatments have remained the same for decades, with significant problems of toxicity, efficacy, lengthy treatments, high costs and the emergence of parasite resistance, in addition to the difficulty of access for the majority of the affected population. All of these reasons support the fact that the development of innovative, effective and safe drugs to fight infections caused by these parasites remains a challenge [3,4].

Leishmaniasis occurs in three clinical forms depending on the location of the parasite in the tissues: visceral, cutaneous and mucocutaneous [5,6]. The most severe form is visceral leishmaniasis, characterized by fever, splenomegaly, hepatomegaly and weight loss, which can be lethal [7]. Cutaneous leishmaniasis presents with skin lesions, the extent and appearance of which depend on the *Leishmania* species and the immune response of the host. The mucocutaneous form of the disease affects the oral mucosa and upper respiratory tract and can lead cause tissue destruction and severe disfigurement [8]. Leishmaniasis affects 12 million people, and the number of new cases is estimated at 2 million each year. In addition, 350 million people who live in endemic areas are at risk of infection [6,9].

Chagas disease presents two clinical phases, one acute and the other chronic. The acute phase lasts from 4 to 8 weeks, and it usually goes unnoticed. It may be asymptomatic or present as a slight fever. The chronic phase, in most cases, remains indeterminate because the patient has no clinical symptoms [10]. However, in 20–30% of cases, several years after the onset of the chronic phase, the determinate form develops, with irreversible damage to the autonomic nervous system in the heart, esophagus or colon [11]. WHO estimates that 10 million people are infected and 90 million people are at risk [12].

Because of the high number of infected people, the severe symptomatology produced, the number of people at risk and the limitations of current treatments, we are required to discover new scaffolds for the development of antiprotozoal drugs against *Leishmania* spp. and *Trypanosoma cruzi* to improve current treatments [13]. These new treatments must provoke programmed cell death (PCD) or apoptotic cell death in the parasite, as this eliminates the exacerbated inflammatory response that produces necrotic cell death and could cause serious damage to the patient [14]. In the case of kinetoplastids, this type of PCD has been described previously, and some very characteristic cellular events occur which include chromatin condensation, oxidative stress and decreased mitochondrial membrane potential, among others [15,16].

Sesquiterpene lactones (SLs) are a large family of secondary metabolites that belong to the group of C15 terpenoids characterized by containing a lactone ring [17,18]. This group of natural compounds is frequently found across the terrestrial plant kingdom, the most common in the family Asteraceae [19]. SLs are also produced by marine organisms, such as fungi [20,21,22]; ciliates [23]; macroalgae [24]; and invertebrates, including sponges [25,26] and corals [27,28,29].

SLs are one of the most prevalent and biologically significant classes of secondary metabolites. Several studies have focused on the relevance and unique aspects of their biological properties, including antiproliferative potential [30]; anti-inflammatory capacity [31]; and potential of antibacterial, antimicrobial and antiviral agents [32]. Additionally, many SLs have exhibited promising effects against protozoan parasites [33,34].

**Figure 1:**
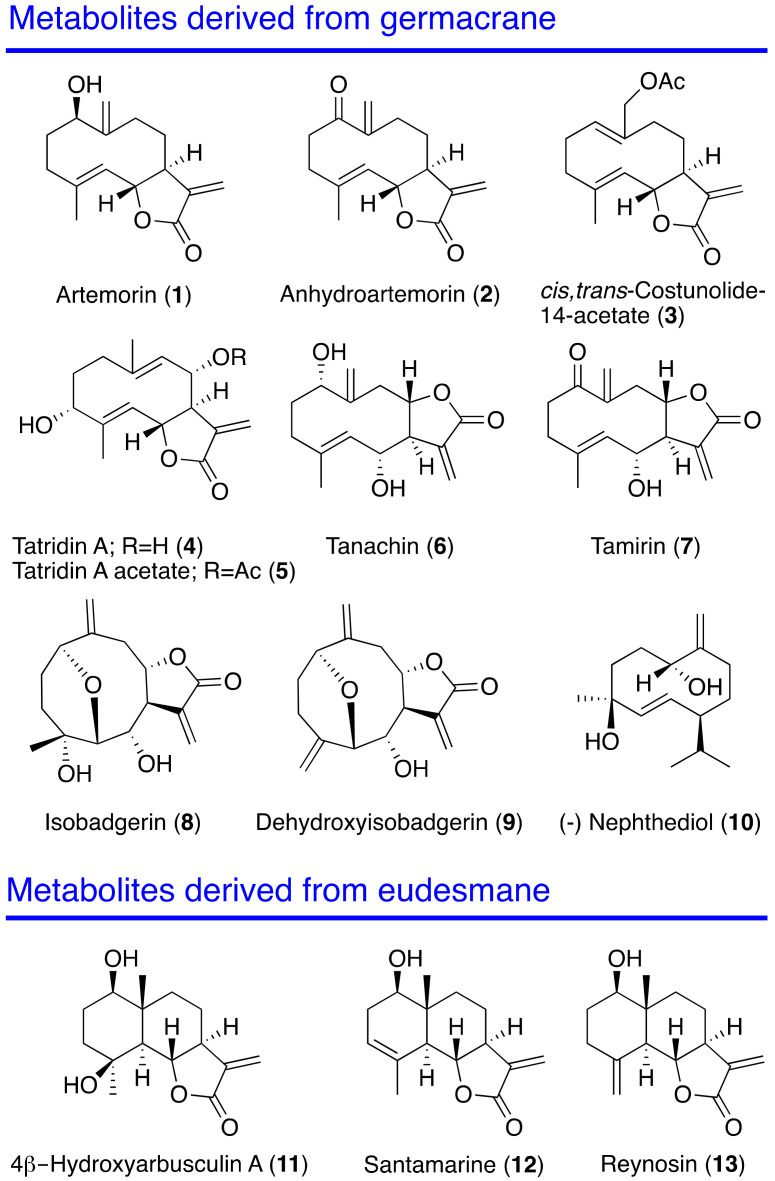
Sesquiterpene compounds isolated from *Palythoa aff. clavata*.

As part of a screening program to identify new marine natural products/scaffolds with antikinetoplastid properties, the chemical constituents of specimens of the zoanthid *Palythoa aff. clavata* collected off the coast of the island of Lanzarote, Canary Islands, were analyzed [33,35] (Figure 1). A study of the activity against *Leishmania* and *Trypanosoma* species was carried out.

## 2. Results and Discussion

### 2.1. Source of Natural Metabolites

A collection of thirteen sesquiterpenes derived from germacrene (**1**–**10**) and from eudesmane (**11**–**13**) were identified from the organic extract of specimens of the zoanthid *Palythoa aff. clavata* collected in the Canary Islands. Size-exclusion and RP18 medium pressure chromatography, followed by HPLC, were used to obtain the pure substances [33].

### 2.2. In Vitro Activity

#### 2.2.1. Leishmanicidal and Trypanocidal Activity

All the isolated sesquiterpenoids (**1**–**13**) were analyzed in a biological screening against the antikinetoplastid species: promastigotes and amastigotes of *Leishmania amazonensis*, promastigotes of *L. donovani* and epimastigotes of *Trypanosoma cruzi*. The results are summarized in Table 1.

The IC_50_ values of all tested compounds against the different parasites ranged between 4.13 and 201.35 μM for promastigotes of *L. donovani,* between 8.62 and 105.32 μM for *L. amazonensis* promastigotes and from 7.44 to 40.13 μM when tested against amastigotes of *L. amazonensis.* In the case of epimastigotes of *T. cruzi*, the activity values ranged from 8.88 to 201.35 μM.

Germacranes **3** and **7** were the most active compounds against promastigotes of both *leishmania* species at 5.41 ± 1.28 and 4.13 ± 1.25 μM, respectively, whereas amastigotes of *L. amazonensis* showed to be more sensitive when treated with compounds anhydroartemorin (**2**) (7.44 ± 0.45 μM) and 4b-Hydroxyarbusculin A (**11**) (15.81 ± 1.01 μM). On the other hand, **2** and **3** showed to be the most active compounds against *T. cruzi* together with the eudesmane sesquiterpene santamarine (**12**) at 13.01 ± 4.95, 8.88 ± 0.91, and 9.74 ± 2.68 μM, respectively. However, none of the active compounds improved the activity values shown by the reference drugs against all tested strains.

#### 2.2.2. Cytotoxic Activity and Selectivity

The cytotoxicity of the sesquiterpenoids **1–13** was tested against murine macrophages J744A.1 to determine 50% cytotoxic concentration (CC_50_). The results are presented in Table 1, showing values of CC_50_ that range between 19.29 and 402.71 μM. Among the compounds that showed better antikinetoplastid effects (**2**, **3**, **7**, **11** and **12**), tamirin (**7**) was the most toxic at 23.32 ± 1.27 μM, while anhydroartemorin (**2**), with a CC_50_ of 179.01 ± 2.27 μM, and 4b-Hydroxyarbusculin A (**11**), with a CC_50_ above 402.71 μM, were the least toxic compounds tested.

To correlate the cytotoxicity with the antiparasitic activity, the selectivity index (SI) was determined and added to Table 2. The analysis of the selectivity index (SI), calculated as the ratio between the cytotoxicity and the antikinetoplastid activity (CC_50_/IC_50_), revealed that anhydroartemorin (**2**) (SI 24.06) and 4β-hydroxyarbusculin A (**11**) (SI > 25.47) displayed slightly improved values to those shown by the reference treatment miltefosine (SI 23.17) against amastigotes of *L. amazonensis*.

Therefore, sesquiterpene lactones **2** and **11** were selected to continue the studies on the mechanisms of action against *L. amazonensis*. Due to the positive antikinetoplastid effects shown against *L. donovani*, the germacrene *cis-trans*-costunolide-14-acetate (**3**) was also included in this study. Anhydroartemorin (**2**) was the selected sesquiterpene lactone to study the mechanisms of action against *T. cruzi*.

### 2.3. Mechanisms of Action of Compounds 2, 3 and 11

Studies on chromatin condensation, plasmatic membrane permeability, ATP levels, mitochondrial membrane potential and detection of reactive oxygen species (ROS) were performed with the aim to identify the cellular effects caused by the active sesquiterpenes **2**, **3** and **11** on the parasites.

#### 2.3.1. Chromatin Condensation Analysis

The occurrence of chromatin condensation in the treated parasites was measured by using the Vybrant^®^ Apoptosis Assay Kit n°5 (Invitrogen™, Thermo Fisher Scientific, Waltham, MA, USA). The kit consists of two reagents: Hoechst and propidium iodide (PI). Hoechst (350⁄461 nm excitation/emission) is a reagent that is capable of crossing membranes and induces blue fluorescence in contact with condensed chromatin; thus, when it is in contact with viable cells, it produces a faint blue fluorescence, and when in contact with cells undergoing apoptosis, it induces an intense blue fluorescence. Propidium iodide (535⁄617 nm excitation/emission), in contrast to Hoechst, only penetrates cells when the plasma membrane is disrupted, producing a red fluorescence in the case of cells undergoing cell death. Intense blue fluorescence accompanied by red indicates late apoptosis, whereas no blue fluorescence in the presence of red indicates necrotic cell death. 

**Figure 2:**
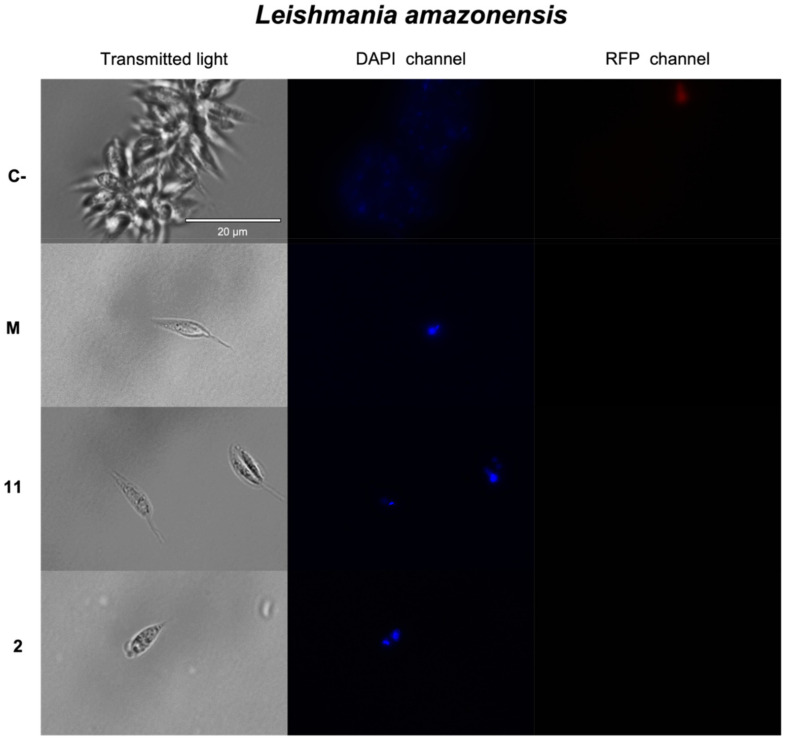

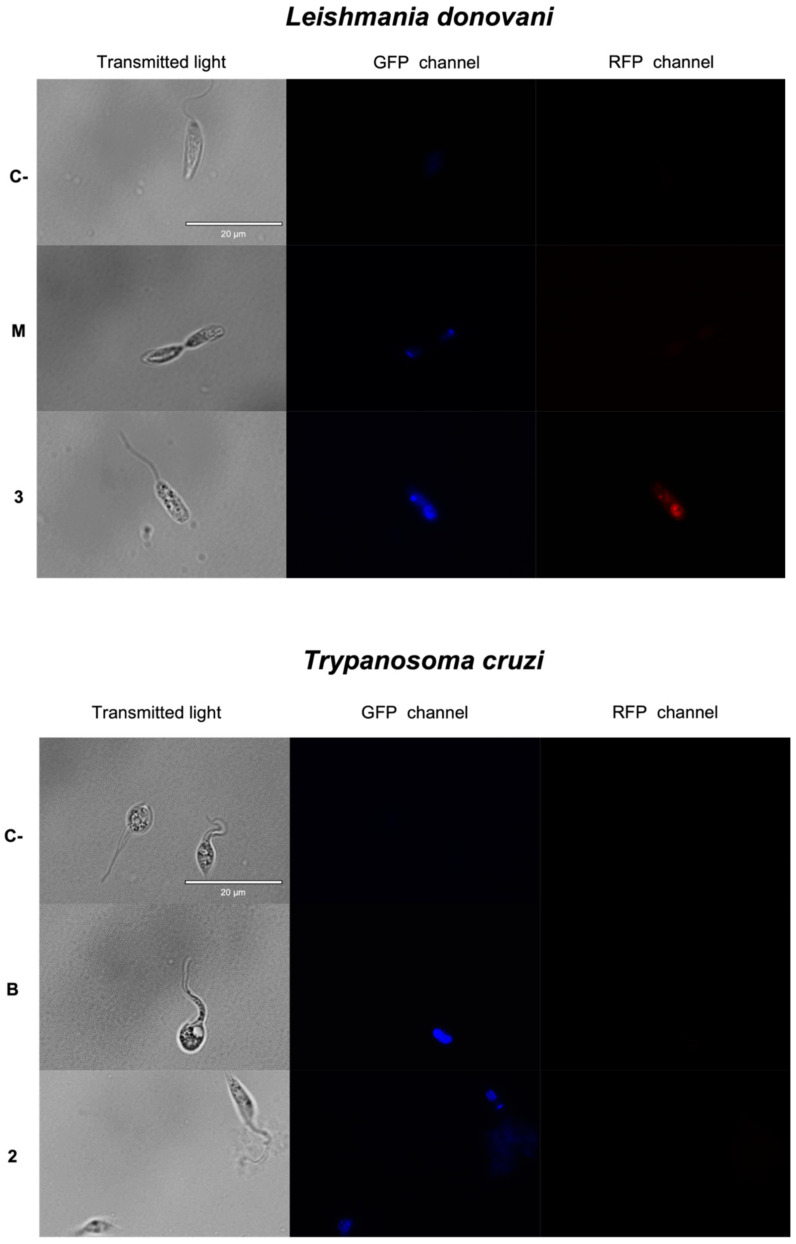
Detection of chromatin condensation by Vybrant^®^ Apoptosis Assay Kit n°5, Hoechst 33342/Propidium Iodide. The images represent promastigotes of *L. amazonensis* after 24 h of incubation with the IC_90_ of 4β-hydroxyarbusculin A (**11**) and anhydroartemorin (**2**), promastigotes of *L. donovani* after 24 h of incubation with the IC_90_ of *cis,trans*-costunolide-14-acetate (**3**) and epimastigotes of *T. cruzi* after 24 h of incubation with the IC_90_ of anhydroartemorin (**2**). Parasites without any treatment were used as negative control (**C-**), and miltefosine (**M**) and benznidazole (**B**) were used as reference treatments against *Leishmania* spp. and *Trypanosoma cruzi*, respectively. Pictures were captured by using an EVOS FL Cell Imaging System (100×). Scale bar: 20 μm.

The results showed that all three compounds produced blue fluorescence in the kinetoplastids studied when incubated at the IC_90_ for 24 h. Anhydroartemorin (**2**) and 4β-Hydroxyarbusculin A (**11**) induced chromatin condensation in *L. amazonensis*, whereas, in the case of *cis,trans*-costunolide-14-acetate (**3**), blue and red fluorescence was produced in promastigotes of *L. donovani*, indicative of a late apoptotic cell death. Anhydroartemorin (**2**) also caused chromatin condensation in *T. cruzi* (Figure 2).

#### 2.3.2. Plasmatic Membrane Permeability Analysis

**Figure 3:**
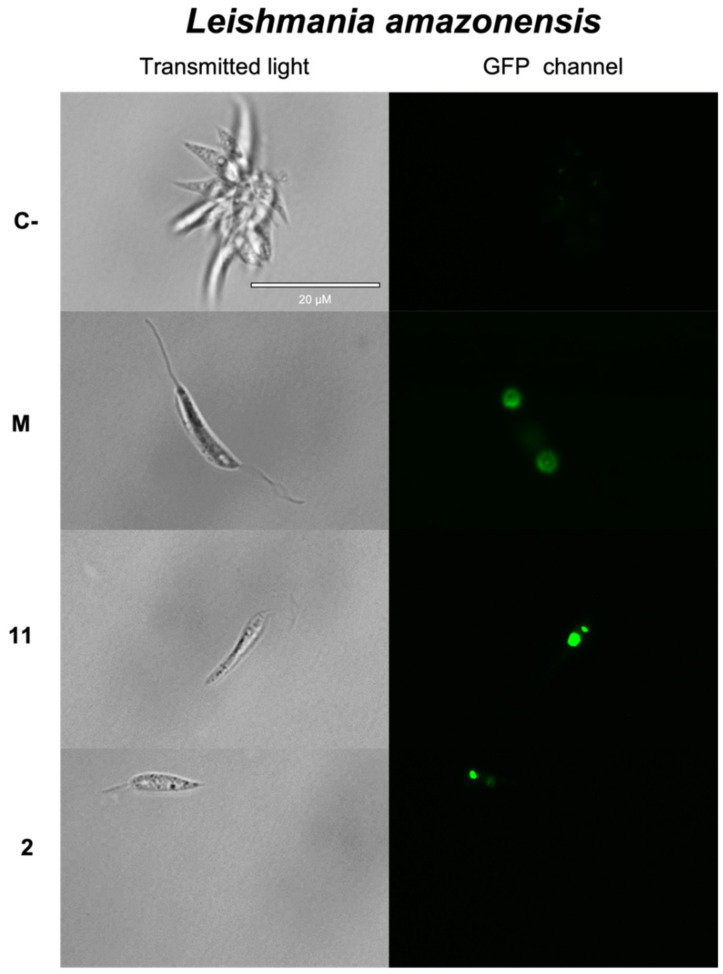

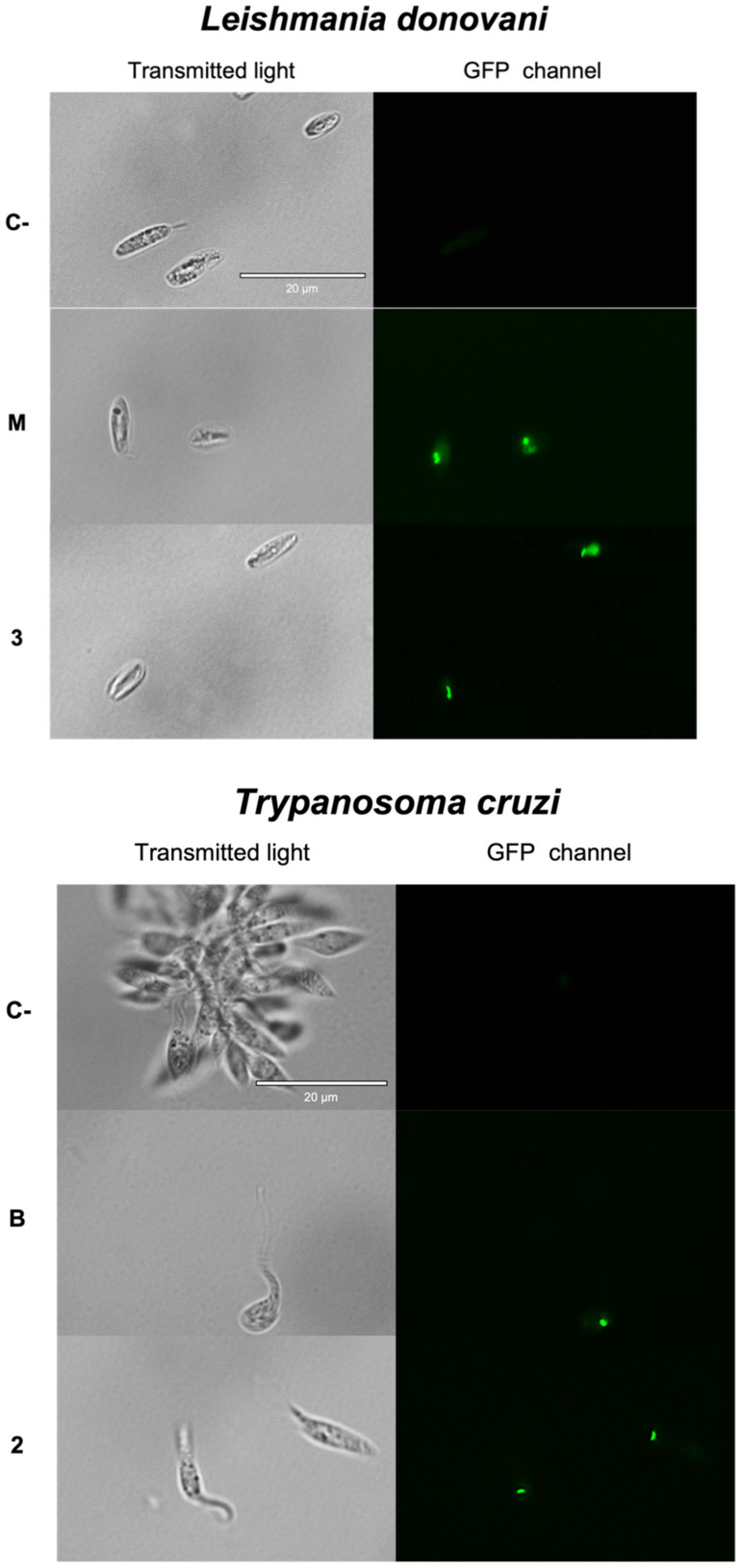
Detection of changes on the plasmatic membrane permeability by SYTOX^®^ Green staining. The images represent promastigotes of *L. amazonensis* after 24 h of incubation with the IC_90_ of 4β-hydroxyarbusculin A (**11**) and anhydroartemorin (**2**), promastigotes of *L. donovani* after 24 h of incubation with the IC_90_ of *cis,trans*-costunolide-14-acetate (**3**) and epimastigotes of *T. cruzi* after 24 h of incubation with the IC_90_ of anhydroartemorin (**2**). Parasites without any treatment were used as negative control (**C-**), and miltefosine (**M**) and benznidazole (**B**) were used as reference treatments against *Leishmania* spp. and *Trypanosoma cruzi*, respectively. Pictures were captured by using an EVOS FL Cell Imaging System (100×). Scale bar: 20 μm.

To analyze alterations on the permeability of the plasmatic membrane, we used the reagent SYTOX^®^ Green. The interaction of SYTOX^®^ Green with the DNA of the studied kinetoplastids emits an intense green fluorescence that can be captured with a fluorescence microscopy (absorption at 502 nm and emission at 523 nm). The incubation of the parasites with the IC_90_ of compounds for 24 h produced green fluorescence inside the nuclei in all cases. Hence, the sesquiterpenes tested caused an increase on the plasmatic membrane permeability of the kinetoplastids studied: anhydroartemorin (**2**) and 4β-hydroxyarbusculin A (**11**) against *L. amazonensis, cis-trans*-costunolide-14-acetate (**3**) on *L. donovani* and anhydroartemorin (**2**) when incubated with *T. cruzi* (Figure 3).

#### 2.3.3. ATP Levels Analysis

**Figure 4:**
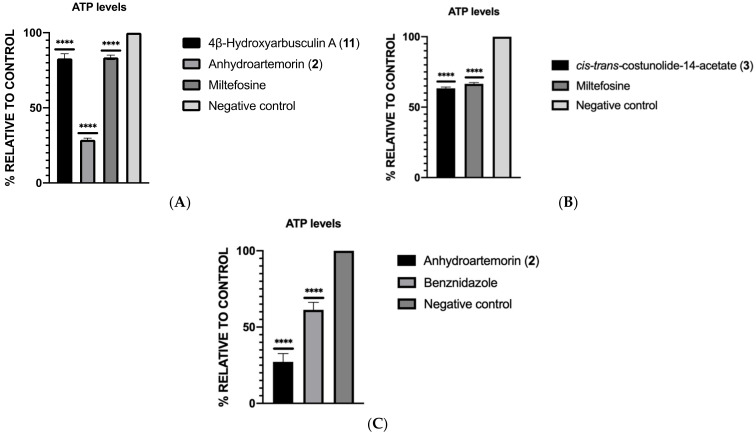
Percentage of ATP relative to the negative control in *L. amazonensis* (**A**), *L. donovani* and (**B**) and *T. cruzi* (**C**). A one-way ANOVA by GraphPad.PRISM^®^ 9.0 software was performed to know the statistical differences between compounds and untreated control (*p* < 0.0001 [****]) (*n* = 3).

The levels of ATP were measured by using CellTiter-Glo^®^ reagent, and values of luminescence were obtained by the EnSpire Multimode Plate Reader^®^. Results were processed and represented in Figure 4 as the percentage of ATP relative to the negative control (without treatment) that corresponds with 100% of ATP level. The results showed that all the sesquiterpenes significantly induced lower ATP levels on the parasites than the negative control. The most remarkable decrease in the ATP level was produced by anhydroartemorin (**2**) in two of the studied parasites, decreasing its quantity by more than 70% on *L. amazonensis* (28.76%) and *T. cruzi* (27.17%).

#### 2.3.4. Mitochondrial Membrane Potential Analysis

**Figure 5:**
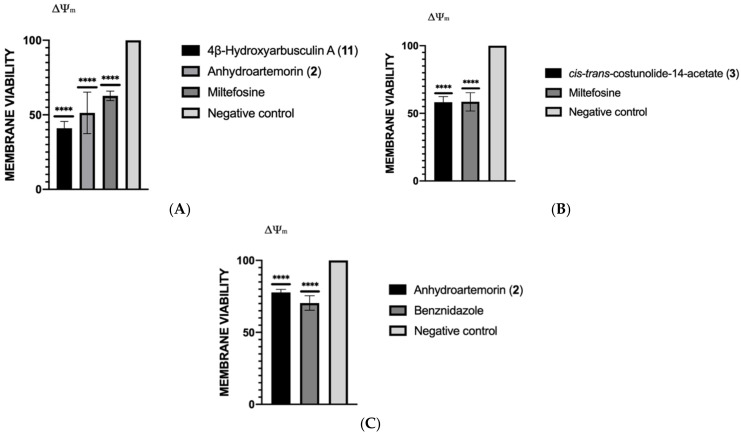
Mitochondrial membrane potential relative to the negative control in *L. amazonensis* (**A**), *L. donovani* (**B**) and *T. cruzi* (**C**). A one-way ANOVA by GraphPad.PRISM^®^ 9.0 software was performed to know the statistical differences between compounds and untreated control (*p* < 0.0001 [****]) (*n* = 3).

The alterations on the mitochondrial membrane potential were measured by using JC-1^®^ reagent. The results were expressed in percentage of variations in the mitochondrial membrane potential relative to the untreated control. The results showed that all the sesquiterpenes studied produced significant lower values of mitochondrial membrane potential than the negative control (without treatment) (Figure 5). The most remarkable difference was observed in the case of 4β-hydroxyarbusculin A (**11**) when incubated with *L. amazonensis* (40.99%).

#### 2.3.5. Reactive Oxygen Species (ROS) Detection

The presence of oxidative stress was measured by using CellROX^®^ Deep Red Reagent. This reagent produces an intense red fluorescence in the presence of ROS detected by fluorescence microscopy (absorption, 485 nm; emission, 520 nm). 

**Figure 6:**
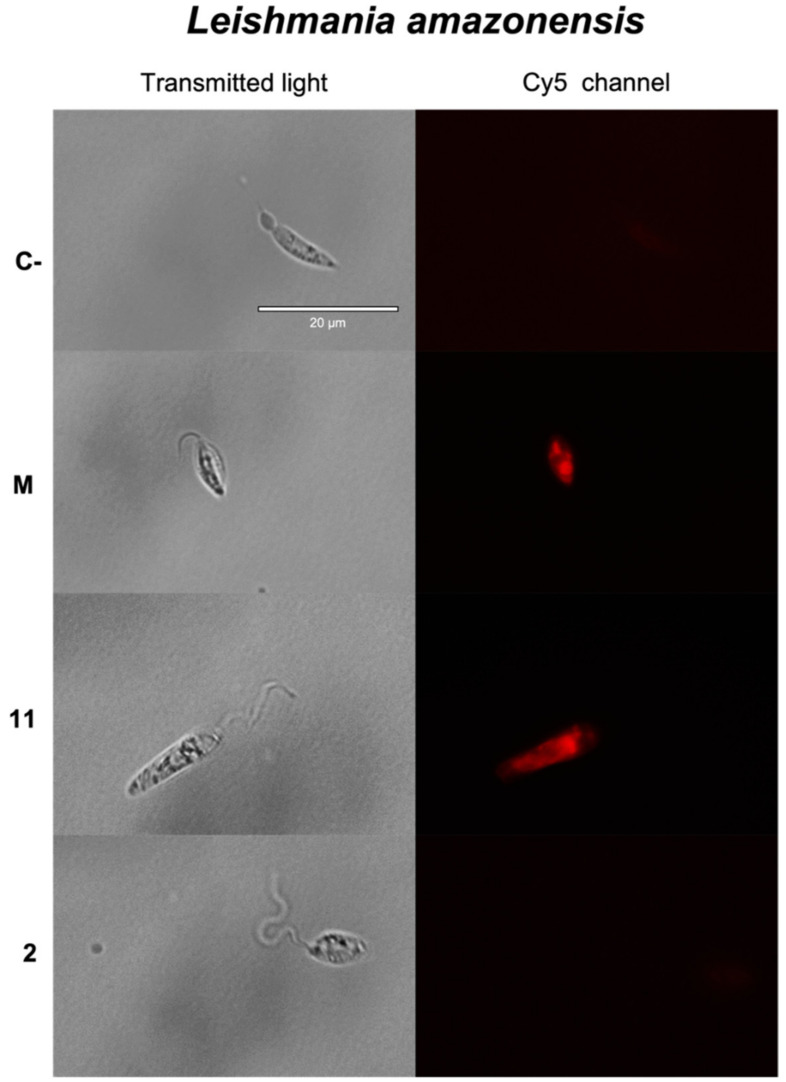

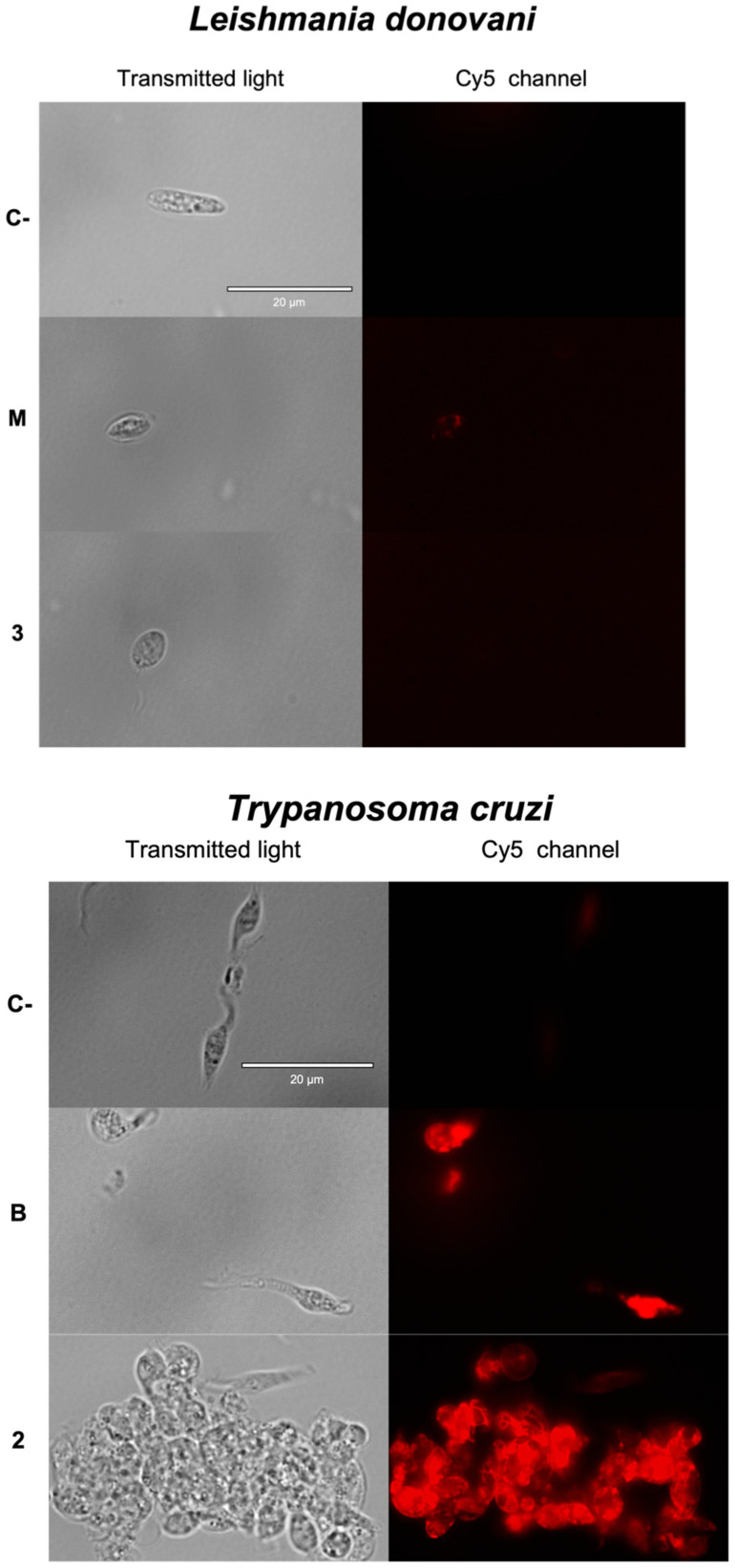
Detection of reactive oxygen species by CellROX^®^ Deep Red Reagent. The images represent promastigotes of *L. amazonensis* after 24 h of incubation with the IC_90_ of 4β-hydroxyarbusculin A (**11**) and anhydroartemorin (**2**), promastigotes of *L. donovani* after 24 h of incubation with the IC_90_ of *cis,trans*-costunolide-14-acetate (**3**) and epimastigotes of *T. cruzi* after 24 h of incubation with the IC_90_ of anhydroartemorin (**2**). Parasites without any treatment were used as negative control (**C-**), and miltefosine (**M**) and benznidazole (**B**) were used as reference treatments against *Leishmania* spp. and *Trypanosoma cruzi*, respectively. Pictures were captured by using an EVOS FL Cell Imaging System (100×). Scale bar: 20 μm.

In the case of *Leishmania* spp. (Figure 6), the presence of red fluorescence was only observed after treatment of *L. amazonensis* with the IC_90_ of 4β-hydroxyarbusculin A (**11**) (Figure 6). The results showed an intense fluorescence due to ROS detection in epimastigotes of *T. cruzi* treated with the sesquiterpenoid anhydroartemorin (**2**) (Figure 6). 

### 2.4. Structure–Activity Relationship

**Figure 7:**
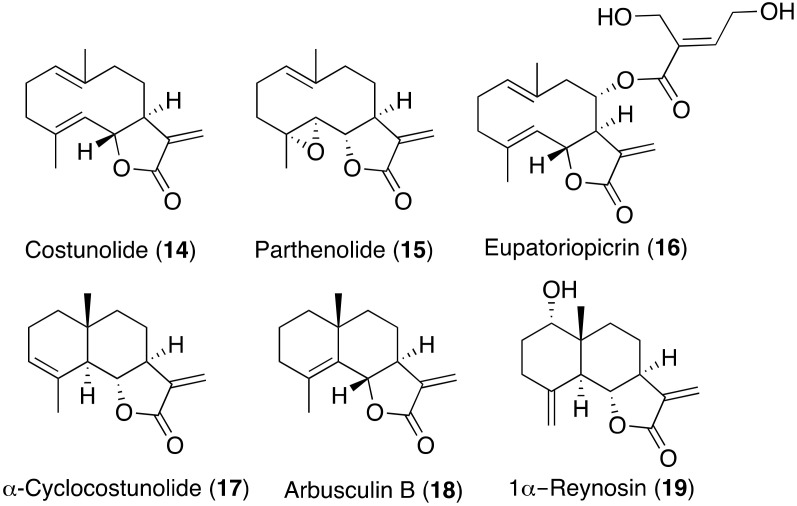
Sesquiterpene lactones from terrestrial sources with reported antikinetoplastid activity.

Sesquiterpene lactones from terrestrial plants have been tested against different kinetoplastids. Thus, from the screening of a large library of plant extracts, the ethyl acetate extract of *Saussurea costus* roots potently inhibited the growth of *Trypanosoma brucei rhodesiense* [36]. The sesquiterpene lactones costunolide (**14**), parthenolide (**15**), eupatoriopicrin (**16**), α-cyclocostunolide (**17**) and arbusculin B (**18**) were identified in the active fractions (Figure 7). They were tested for in vitro anti-trypanosomal activities and cytotoxicity. These compounds exhibited an antiparasitic effect at IC_50_ values ranging between 0.8 and 22 μM against *T. brucei rhodesiense*, showing a cytotoxic effect against L6 cells at CC_50_ from 1.6 to 19 μM, and selectivity indexes from 0.5 to 6.5. Parthenolide (**15**) has been also reported as antiprotozoal substance against *T. cruzi* at IC_50_ 2.01 μM [37]. Within the eudesmane-related compounds, the antikinetoplastid properties of 1a- and 1b-reynosin have been reported by Schmidt et al., with compound **19** showing a high selectivity against *T. brucei rhodes* [38].

**Figure 8:**
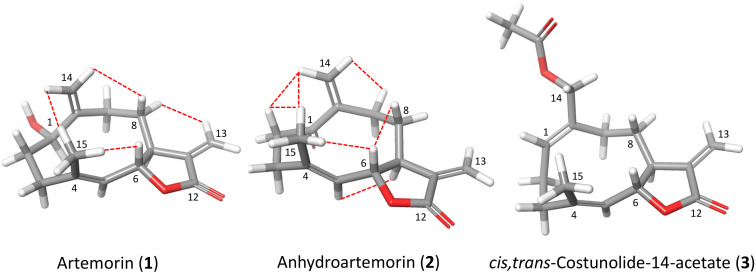
Three-dimensional chemical structure of artemorin (**1**), anhydroartemorin (**2**) and *cis,trans*-costunolide-14-acetate (**3**). Selected NOE correlations are indicated in dotted red lines for (**1**) and (**2**).

In the marine sesquiterpene series reported here, eight out of all tested sesquiterpene compounds showed activity against the parasites revealing a different effectiveness depending on the kinetoplastid species. Anhydroartemorin (**2**) and tamirin (**7**) were among the most active compounds. The comparison of the chemical structure of artemorin (**1**) vs. anhydroartemorin (**2**) or tanachin (**6**) vs. tamirin (**7**) show that the presence of an additional α,β-unsaturated carbonyl system, which acts as Michael acceptors, increases the antiparasitic activity and the cytotoxicity (Figure 1 and Figure 8). Another important parameter that influences the structure–activity relationship is liposolubility [39]. Thus, for the germacranolide series, *cis,trans*-costunolide-14-acetate (**3**) is the most hydrophobic compound with a CLogP of 2.949.

**Figure 9:**
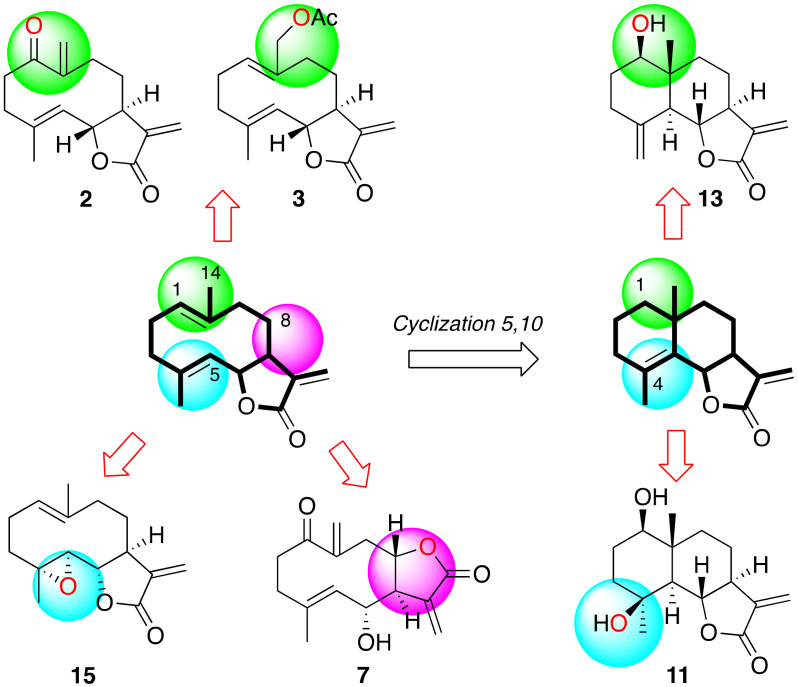
Structure–activity relationship of germanane and eudesmane metabolites against kinetoplastid parasites.

Thus, among the sesquiterpene lactones isolated from *Palythoa aff. clavata*, it possible to observe that germacranolides functionalized at positions C-1 and C-14 are those that present the most interesting antiparasitic activity. This bioactivity is reinforced by the existence of a second Michael acceptor, as is the case of anhydroartemorin (**2**) or tamirin (**7**) (Figure 9). From previous works carried out with sesquiterpene lactones of terrestrial origin, it was also observed that compounds with functionalization at carbons C-4 and C-5, such as parthenolide (**15**), were effective for the activity against kinetoplastids. Similarly, in the eudesmanolide series, functionalization at position C-4 results in being essential for the antiparasitic activity, with compound **11** as the most effective of the three tested samples. On the other hand, the lactone cyclization at carbon C-6 or carbon C-8 does not imply a significant alteration of the activity.

In summary, seven out of the thirteen sesquiterpenoids studied in this work presented good values of IC_50_ against the promastigotes stage of *L. amazonensis*, four against promastigotes of *L. donovani* and six against epimastigotes of *T. cruzi.* In the case of the intracellular form of the parasite, four sesquiterpenoids presented good activity against amastigotes of *L. amazonensis.*

Regarding the cytotoxicity, the best selectivity indexes obtained were *cis,trans*-costunolide-14-acetate (**3**) against promastigotes of *L. donovani* and anhydroartemorin (**2**) against *T. cruzi*. In the case of amastigotes of *L. amazonensis*, the most selective products were anhydroartemorin (**2**) and 4β-hydroxyarbusculin A (**11**). These most selective compounds were chosen to determine the mechanism of action that these sesquiterpenoids could induce in the parasites. 

It is important to mention that sesquiterpene lactones **2**, **3** and **11** seem to induce an apoptotic-like death or programmed cell death (PCD) in the kinetoplastids, and therefore could contribute to eliminate the inflammation problems caused by a necrotic process.

Comparing these results with those previously obtained in the literature, we can observe similarities. It has been reported that sesquiterpenes can develop numerous activities, such as anti-inflammatory, antiviral or antibacterial activities [31,32]. However, their activity as antiparasitic has also been reported, with activity against free-living amoebae [33,40], or their activity against kinetoplastids, already reported previously by other sesquiterpenes [41,42].

On the other hand, the involvement of sesquiterpenes in PCD has also been previously mentioned in the literature as being in plant cells, such as *Aquilaria sinensis* [43]; or in parasites, such as *Naegleria fowleri* [40] and *Acanthamoeba* spp. [33], and even in kinetoplastids, *Leishmania* spp. and *T. cruzi* [41,42].

Similarities in PCD-related events are also observed. An example is the case of *Naegleria fowleri* treated with sesquiterpenes, where DNA condensation, damage in cell membrane and mitochondria and ROS generation can be observed [40]. The same occurs in the cases of *Acanthamoeba* spp. and kinetoplastids, where numerous events related to PCD, alteration of the plasma membrane, decrease of the mitochondrial membrane potential or decrease of cellular ATP levels, among others, are reported [33,41,42].

In conclusion, the sesquiterpenes studied show antikinetoplastid activity against *Trypanosoma cruzi*, *Leishmania amazonensis* and *Leishmania donovani*, as well as programmed cell death induction, which makes them promising treatments for Chagas disease and leishmaniasis.

## 3. Materials and Methods

### 3.1. General Experimental Procedures

The compounds’ separations were carried out with Sephadex LH-20 (Pharmacia Fine Chemicals^®^) prepaid column Lobar^®^ Gröbe B (310-25) LiChroprep^®^ RP-18 (40–63 μm) (Merck Darmstadt, Germany) and HPLC (high-performance liquid chromatography) Agilent 1260 Infinity Quaternary LC equipped with a Diode Array Detector (Waldbronn, Germany). TLC (Thin layer chromatography) (Merck Darmstadt, Germany) silica gel used to monitor column chromatography was visualized by UV light (254 nm) and spraying with cobalt chloride reagent (2% in sulfuric acid 10%) and heating. Chemical characterization of the isolated compounds was determined by 1D and 2D NMR, using a Bruker AVANCE 500 or 600 MHz (Bruker Biospin, Falländen, Switzerland) when required. Bruker AVANCE 600 MHz instrument is equipped with a 5 mm TCI inverse detection cryoprobe (Bruker Biospin, Falländen, Switzerland). Standard Bruker NMR pulse sequences were utilized. NMR spectra were obtained by dissolving samples in CDCl_3_ (99.9%), and chemical shifts are reported relative to solvent (δ_H_ 7.26 and δ_C_ 77.0 ppm). IR spectra were recorded on a Bruker IFS66/S (Ettlingen, Germany) equipped with an ATR accessory, using CHCl_3_ solutions. The EnSpire^®^ Multimode Reader (Perkin Elmer, Waltham, MA, USA) was used to obtain absorbance values of alamarBlue^®^ reagent (Bio-Rad Laboratories, Oxford, UK). HR-ESI–MS data were obtained on a Waters LCT Premier XE Micromass (Manchester, UK) and VG AutoSpec Micromass spectrometers (Manchester, UK), respectively. Optical rotations were measured in CHCl_3_ on a PerkinElmer 241 polarimeter (Waltham, MA, USA) by using a Na lamp.

### 3.2. Biological Material

Polyps of the soft coral *Palythoa aff. clavata* was collected at Caletón Blanco, Orzola, North cost of Lanzarote Island, Canary Islands (29°13′05.8″ N 13°26′36.3″ W). The samples were frozen and transferred to the laboratory. The samples were classified by Dr. Alberto Brito and Dr. Adriana Rodríguez Hernández from the Department of Animal Biology, Edaphology and Geology of University of La Laguna.

### 3.3. Isolation of Sesquiterpene Lactones

The frizzed *P. aff. clavata* polyps were extracted with acetone at room temperature. Solvent was replaced three times. The combined extracts were filtered and concentrated under vacuum to obtain, in the end, a yellow-green oil. First, the resulting acetone extract was chromatographed by gel filtration on a Sephadex LH-20 (7Ø × 50 cm), using CHCl_3_:MeOH:n-hexane (1:1:2) as the mobile phase to afford eight fractions grouped according to their similar chemical content by TLC. The fractions containing sesquiterpene metabolites were subsequently fractionated by medium-pressure chromatography, using a Lobar LiChroprep RP18 column with MeOH:H_2_O (17:3) at 1 mL/min. Final purification was performed on an HPLC with a μ-Porasil column, using n-hexane:EtOAc (1:1) to yield ten pure compounds derived from germacrene, namely artemorin (**1**), anhydroartemorin (**2**), cis,trans-costunolide-14-acetate (**3**), tatridin A (**4**), tatridin A acetate (**5**), tanachin (**6**), tamarin (**7**), isobadgerin (**8**), dehydroxyisobadgerin (**9**) and (−) nephthediol (**10**), and three derived from eudesmane, namely 4β-hydroxyarbusculin A (**11**), santamarine (**12**) and reynosin (**13**) [33]. The physical and 1D NMR spectroscopic data of compounds **1–13** are summarized in the Supplementary Materials. All compounds were dissolved in dimethylsulfoxide (DMSO), vortexed until complete solubilization and stored at −20 °C.

### 3.4. Strains

For the anti-leishmanicidal experiments, promastigotes and amastigotes of *Leishmania amazonensis* strain (MHOM/BR/77/LTB0016) and promastigotes of *Leishmania donovani* strain (MHOM/IN/90/GE1F8R) were used, and they were maintained in RPMI 1640 medium (Gibco, Waltham, MA, USA) at 26 °C. For the trypanocidal experiments, epimastigotes of *Trypanosoma cruzi* (Y strain) were used, and they were maintained in liver infusion tryptose (LIT) medium supplemented with 10% of fetal bovine serum (FBS) at 26 °C. Murine macrophages J774A.1 (ATCC #TIB-67), maintained in DMEM medium at 37 °C with 5% CO_2_, were used for the cytotoxic activity assays.

### 3.5. Anti-Kinetoplastid Activity

For the activity against extracellular parasites of kinetoplastid, *Leishmania* and *Trypanosoma* parasites, in a 96-well plate, serial dilutions of the compounds were added with dilutions of 10^5^ parasites per well and a 10% of alamarBlue Cell Viability Reagent^®^ (Thermo Fisher Scientific, Waltham, MA, USA). After 72 h of incubation at 26 °C, the fluorescence of each well was measured in the EnSpire Multimode Plate Reader^®^ (PerkinElmer, Thermo Fisher Scientific, Madrid, Spain). The inhibitory concentration 50 (IC_50_) was calculated by nonlinear regression analysis.

To determinate the IC_50_ against the intracellular form of the parasite amastigotes of *L. amazonensis*, the alamarBlue colorimetric method was also used. In a 96-well plate, murine macrophages were added in a concentration of 10^4^ cells per well. After adherence of the cells, 10^5^ promastigotes of the parasite were added to reach 1:10 cell:parasites per well. After 24 h of incubation at 37 °C with 5% of CO_2_ for the internalization of the parasite, the wells were washed, and serial dilutions of the compounds were added. After an incubation of 24 h with the compounds, the wells were washed, and the macrophages were broken with a solution of SDS (Sodium dodecyl sulfate) at 0.05% for 30 s. After adding fresh RPMI medium, we added 10% of alamarBlue, and the plates were incubated at 26 °C for 96 h. Finally, the fluorescence was measured by using the EnSpire Multimode Plate Reader^®^ (PerkinElmer, Thermo Fisher Scientific, Madrid, Spain), and the IC_50_ of each compound was calculated [44].

### 3.6. Cytotoxic Activity

To determine the cytotoxicity concentration 50 (CC_50_), the same colorimetric assay based on the alamarBlue reagent was used. In a 96-well plate, 10^4^ cells per well were added. When the cells were adhered to the wells, serial dilutions of the compounds and 10% of alamarBlue reagent were added. After an incubation of 24 h at 37 °C with 5% of CO_2_, the fluorescence was determined, and the CC_50_ was calculated. Finally, the selectivity index of each compound was calculated (CC_50_/IC_50_) [45].

### 3.7. Chromatin Condensation Analysis

To determine the presence of chromatin condensation in the treated parasites Vybrant^®^ Apoptosis Assay Kit n°5, we used Hoechst 33342/Propidium Iodide (ThermoFisher Scientific, MA, USA). After 24 h of incubation at 26 °C of the extracellular forms of the parasites with the inhibitory concentration 90 (IC_90_) of the compounds to test, these solutions were centrifuged (825 g, 10 min, 4 °C) and resuspended in 50 μL of buffer. Then, Hoechst (5 μg/mL) and propidium iodide (PI) (1 μg/mL) were added and incubated for 20 min at 26 °C. To show the results, fluorescence pictures were captured in EVOS^®^ FL Cell Imaging System (ThermoFisher Scientific, MA, USA), using DAPI light cube for Hoechst and RFP light cube for PI [46].

### 3.8. Plasmatic Membrane Permeability Analysis

The alterations in the plasmatic membrane permeability were determined by using SYTOX^®^ Green nucleic acid stain fluorescent dye (ThermoFisher Scientific, MA, USA). After a 24-h incubation of the promastigotes/epimastigotes with the IC_90_ of the compounds to test, the solutions were centrifuged (825 g, 10 min, 4 °C) and resuspended in 50 μL of buffer with the kit, at a 1 μM concentration. Thereafter, fluorescence pictures were captured by using the EVOS^®^ FL Cell Imaging System (ThermoFisher Scientific, MA, USA) with the GFP light cube [47].

### 3.9. ATP Levels Analysis

The levels of ATP in the wells were measured by using the CellTiter-Glo^®^ Luminescent Cell Viability Assay (Promega, WI, USA). In a 96-well plate, the parasites with the IC_90_ of the compounds were incubated for 24 h at 26 °C, centrifuged (825 g, 10 min, 4 °C) and resuspended in 25 μL of buffer. These volumes were transferred to a white plate, and 25 μL of kit was added to each well. After 10 min of incubation at room temperature, the luminescence was determined by using the EnSpire Multimode Plate Reader^®^ (PerkinElmer), and the results were expressed as the percentage of production of ATP relative to the negative control (without any treatment). All of the experiments were performed in triplicate in three different days (*n* = 3) [48].

### 3.10. Mitochondrial Membrane Potential Analysis

The alterations in the mitochondrial membrane potential were determined by using the JC-1 Mitochondrial Membrane Potential Assay Kit^®^ (Cayman Chemical, Ann Arbor, MI, USA). After a 24 h incubation of the extracellular parasites with the IC_90_ of the compounds to test, the solutions were centrifuged (825 g, 10 min, 4 °C) and resuspended in 50 μL of buffer and added to a black plate with 5 μL of the kit and incubated for 30 min at 26 °C. Finally, using the EnSpire Multimode Plate Reader^®^ (PerkinElmer, Thermo Fisher Scientific, Madrid, Spain), we measured the fluorescence, and the results were expressed in the percentage relative to the negative control (without any treatment), using the ratio 595 nm/535 nm (aggregates/monomers). All of the experiments were performed in triplicate in three different days (*n* = 3) [49].

### 3.11. Reactive Oxygen Species (ROS) Detection

To measure the oxidative stress of the cell, the presence of reactive oxygen species (ROS) was used, measured by the commercial kit CellROX^®^ Deep Red Reagent (Thermo Fisher Scientific, MA, USA). The IC_90_ of the compounds and the parasites were added to a 96-well plate. After incubating for 24 h, we performed centrifuging and resuspending in 50 μL of buffer with the CellROX kit, at a 5 μM concentration. Finally, fluorescence pictures were captured by using the EVOS^®^ FL Cell Imaging System (Thermo Fisher Scientific, MA, USA) with the Cy5 light cube [50].

### 3.12. Statistical Analysis

All of the experiments were performed in duplicate in three different days. The data of these three independent experiments are presented as the mean ± standard error (SE), and the data shown are the representative results. Inhibitory and cytotoxicity concentrations (IC_50_ and CC_50_) were calculated by non-linear regression analysis with 95% confidence limits. A paired two-tailed t-test was used for the analysis of the data. Values of *p* < 0.05 were considered significant.

## Figures and Tables

**Table 1:** Effects of compounds **1**–**13** isolated from the coral *Palythoa aff. clavata* against kinetoplastid species and murine macrophages (J744A.1). The IC_50_ results correspond to 72 h of incubation in *T. cruzi* epimastigotes and *Leishmania* spp. promastigotes, and to 24 h of incubation in the case of macrophages and *L. amazonensis* amastigotes. Inhibitory concentrations are reported in μM and miltefosine and benznidazole included as reference treatments. Values represent mean concentrations with each standard deviation.

Compounds	Promastigotes *L. donovani* IC_50_ (μM)	Promastigotes *L. amazonensis* IC_50_ (μM)	Amastigotes *L. amazonensis* IC_50_ (μM)	Epimastigotes *T. cruzi* IC_50_ (μM)	Macrophages J744A.1 CC_50_ (μM)
Artemorin (1)	>201.35	40.87 ± 2.82	40.13 ± 3.44	80.98 ± 11.96	>402.71
Anhydroartemorin (2)	>101.50	25.56 ± 5.88	7.44 ± 0.45	13.01 ± 4.95	179.01 ± 2.27
*cis,trans*-costunolide-14-acetate (3)	5.41 ± 1.28	8.62 ± 1.00	24.57 ± 3.64	8.88 ± 0.91	51.04 ± 7.54
Tatridin A (4)	>94.58	>94.58		>94.58	32.38 ± 6.55
Tatridin A acetate (5)	>81.60	>81.60		>81.60	19.29 ± 0.91
Tanachin (6)	>94.58	>94.58		>94.58	99.24 ± 4.43
Tamirin (7)	4.13 ± 1.25	11.71 ± 2.52		>69.00	23.32 ± 1.27
Isobadgerin (8)	>89.18	>89.18		>89.18	89.68 ± 3.92
Dehydroxyisobadgerin (9)	34.04 ± 3.32	25.54 ± 3.2		59.78 ± 9.76	49.98 ± 13.08
Nephthediol (10)	>105.32	>105.32		>105.32	23.58 ± 0.21
4b-Hydroxyarbusculin A (11)	>201.35	76.39 ± 5.72	15.81 ± 1.01	>201.35	>402.71
Santamarine (12)	12.13 ± 4.67	>100.67		9.74 ± 2.68	77.08 ± 13.97
Reynosin (13)	>201.35	40.19 ± 8.78		58.23 ± 13.13	77.92 ± 13.85
Miltefosine	3.32 ± 0.27	6.48 ± 0.24	3.12 ± 0.30		72.19 ± 3.06
Benznidazole				6.94 ± 1.94	>1536

**Table 2:** Selectivity indexes (SIs) of compounds **1**–**13** isolated from the coral *Palythoa aff. clavata* against kinetoplastid species. The miltefosine and benznidazole were included as reference treatments. ND: not determined.

Compounds	Promastigotes *L. donovani* SI	Promastigotes *L. amazonensis* SI	Amastigotes *L. amazonensis* SI	Epimastigotes *T. cruzi* SI
Artemorin (1)	ND	>9.85	>10.04	>4.97
Anhydroartemorin (2)	<1.76	7.00	24.06	13.76
*cis,trans*-costunolide-14-acetate (3)	9.43	5.92	2.08	5.75
Tatridin A (4)	<0.34	<0.34	ND	<0.34
Tatridin A acetate (5)	<0.24	<0.24	ND	<0.24
Tanachin (6)	<1.05	<1.05	ND	<1.05
Tamirin (7)	5.65	1.99	ND	<0.34
Isobadgerin (8)	<1.01	<1.01	ND	<1.01
Dehydroxyisobadgerin (9)	1.47	1.96	ND	0.84
Nephthediol (10)	<0.22	<0.22	ND	<0.22
4b-Hydroxyarbusculin A (11)	ND	>5.27	>25.47	ND
Santamarine (12)	6.35	<0.77	ND	7.91
Reynosin (13)	<0.39	1.94	ND	1.34
Miltefosine	21.74	11.14	23.17	ND
Benznidazole	ND	ND	ND	221.33

## Data Availability

Data are contained within the article.

## Supplementary Materials

The following are available online at https://www.mdpi.com/article/10.3390/ph14111095/s1, Figure S1: Overlays of the detection of chromatin condensation by Vybrant^®^ Apoptosis Assay Kit n°5, Hoechst 33342/Propidium Iodide, changes on the plasmatic membrane permeability by SYTOX^®^ Green staining, and reactive oxygen species by CellROX^®^ Deep Red Reagent in *Leishmania amazonensis*, Figure S2: Overlays of the detection of chromatin condensation by Vybrant^®^ Apoptosis Assay Kit n°5, Hoechst 33342/Propidium Iodide, changes on the plasmatic membrane permeability by SYTOX^®^ Green staining, and reactive oxygen species by CellROX^®^ Deep Red Reagent in *Leishmania donovani*, Figure S3: Overlays of the detection of chromatin condensation by Vybrant^®^ Apoptosis Assay Kit n°5, Hoechst 33342/Propidium Iodide, changes on the plasmatic membrane permeability by SYTOX^®^ Green staining, and reactive oxygen species by CellROX^®^ Deep Red Reagent in *T. cruzi*, Table S1: Physical and spectroscopic data of compounds **1** and **2** [NMR: CDCl_3_, 500 MHz, 298K], Table S2: Physical and spectroscopic data of compounds **3-5** [NMR: CDCl_3_, 500 MHz, 298K], Table S3: Physical and spectroscopic data of compounds **6** and **7** [NMR: CDCl_3_, 500 MHz, 298K], Table S4: Physical and spectroscopic data of compounds **8** and **9** [NMR: CDCl_3_, 500 MHz, 298K], Table S5: Physical and spectroscopic data of (-)-nephtediol (**10**) [NMR: CDCl_3_, 500 MHz, 298K], Table S6: Physical and spectroscopic data of compounds **11-13** [NMR: CDCl_3_, 500 MHz, 298K].
