# Supplementary material for: Antikinetoplastid Activity of Sesquiterpenes Isolated from the Zoanthid Palythoa aff. clavata
Source: Pharmaceuticals (Basel). 2021 Oct 28;14(11):1095. doi: 10.3390/ph14111095 (PMC8625207; doi:10.3390/ph14111095)
Supplement: Supplementary file 1 [file pharmaceuticals-14-01095-s001.zip › pharmaceuticals-1414645-supplementary.pdf]

Supplementary Material

Antikinetoplastid activity of sesquiterpenes isolated from the zoanthid *Palythoa aff. clavata*

Carlos J. Bethencourt-Estrella[1,2,3], Nathalia Nocchi[4,5], Atteneri López-Arencibia[1,2,3], Desirée San Nicolás-Hernández[1,2,3], María L. Souto[4,5], Blanca Suárez-Gómez[4], Ana R. Díaz-Marrero[4,*], José J. Fernández[4,5,*], Jacob Lorenzo-Morales[1,2,3,6*] and José E. Piñero[1,2,3,6*]

Table of Contents

CONTENTS		Page
Figure S1	Overlays of the detection of chromatin condensation by Vybrant® Apoptosis Assay Kit n°5, Hoechst 33342/Propidium Iodide, changes on the plasmatic membrane permeability by SYTOX® Green staining, and reactive oxygen species by CellROX® Deep Red Reagent in *Leishmania amazonensis.*	3
Figure S2	Overlays of the detection of chromatin condensation by Vybrant® Apoptosis Assay Kit n°5, Hoechst 33342/Propidium Iodide, changes on the plasmatic membrane permeability by SYTOX® Green staining, and reactive oxygen species by CellROX® Deep Red Reagent in *Leishmania donovani.*	4
Figure S3	Overlays of the detection of chromatin condensation by Vybrant® Apoptosis Assay Kit n°5, Hoechst 33342/Propidium Iodide, changes on the plasmatic membrane permeability by SYTOX® Green staining, and reactive oxygen species by CellROX® Deep Red Reagent in *T. cruzi.*	5
Table S1	Physical and spectroscopic data of compounds **1** and **2** [NMR: $CDCl_3$, 500 MHz, 298K]	6
Table S2	Physical and spectroscopic data of compounds **3-5** [NMR: $CDCl_3$, 500 MHz, 298K]	7
Table S3	Physical and spectroscopic data of compounds **6** and **7** [NMR: $CDCl_3$, 500 MHz, 298K]	8
Table S4	Physical and spectroscopic data of compounds **8** and **9** [NMR: $CDCl_3$, 500 MHz, 298K]	9
Table S5	Physical and spectroscopic data of (-)-nephtediol (**10**) [NMR: $CDCl_3$, 500 MHz, 298K]	10

**Table S6**	Physical and spectroscopic data of compounds **11-13** [NMR: $CDCl_3$, 500 MHz, 298K]	11

Figure S1. Overlays of the detection of chromatin condensation by Vybrant® Apoptosis Assay Kit n°5, Hoechst 33342/Propidium Iodide, changes on the plasmatic membrane permeability by SYTOX® Green staining, and reactive oxygen species by CellROX® Deep Red Reagent in *Leishmania amazonensis*.

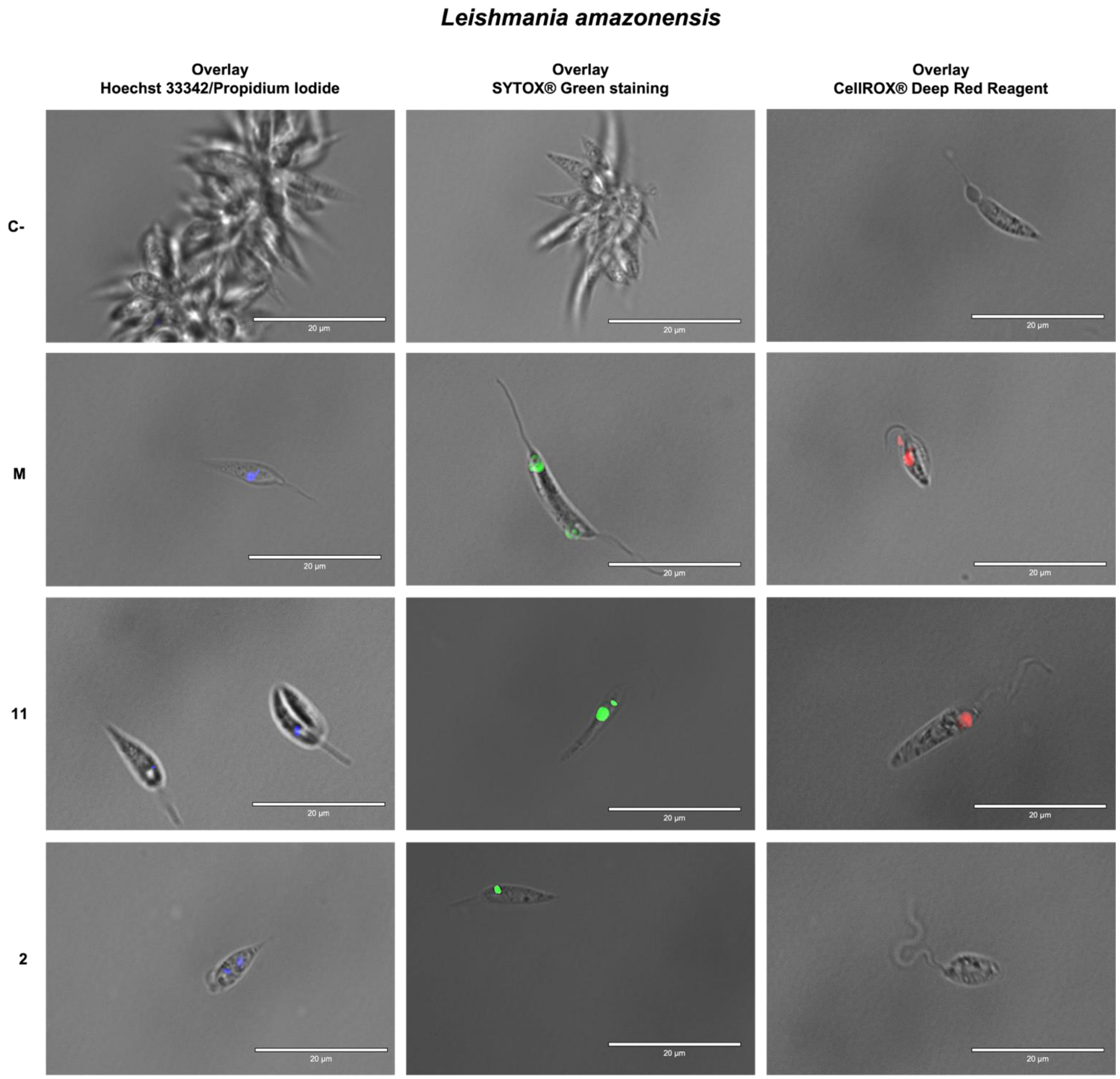

Figure S2. Overlays of the detection of chromatin condensation by Vybrant® Apoptosis Assay Kit n°5, Hoechst 33342/Propidium Iodide, changes on the plasmatic membrane permeability by SYTOX® Green staining, and reactive oxygen species by CellROX® Deep Red Reagent in *Leishmania donovani.*

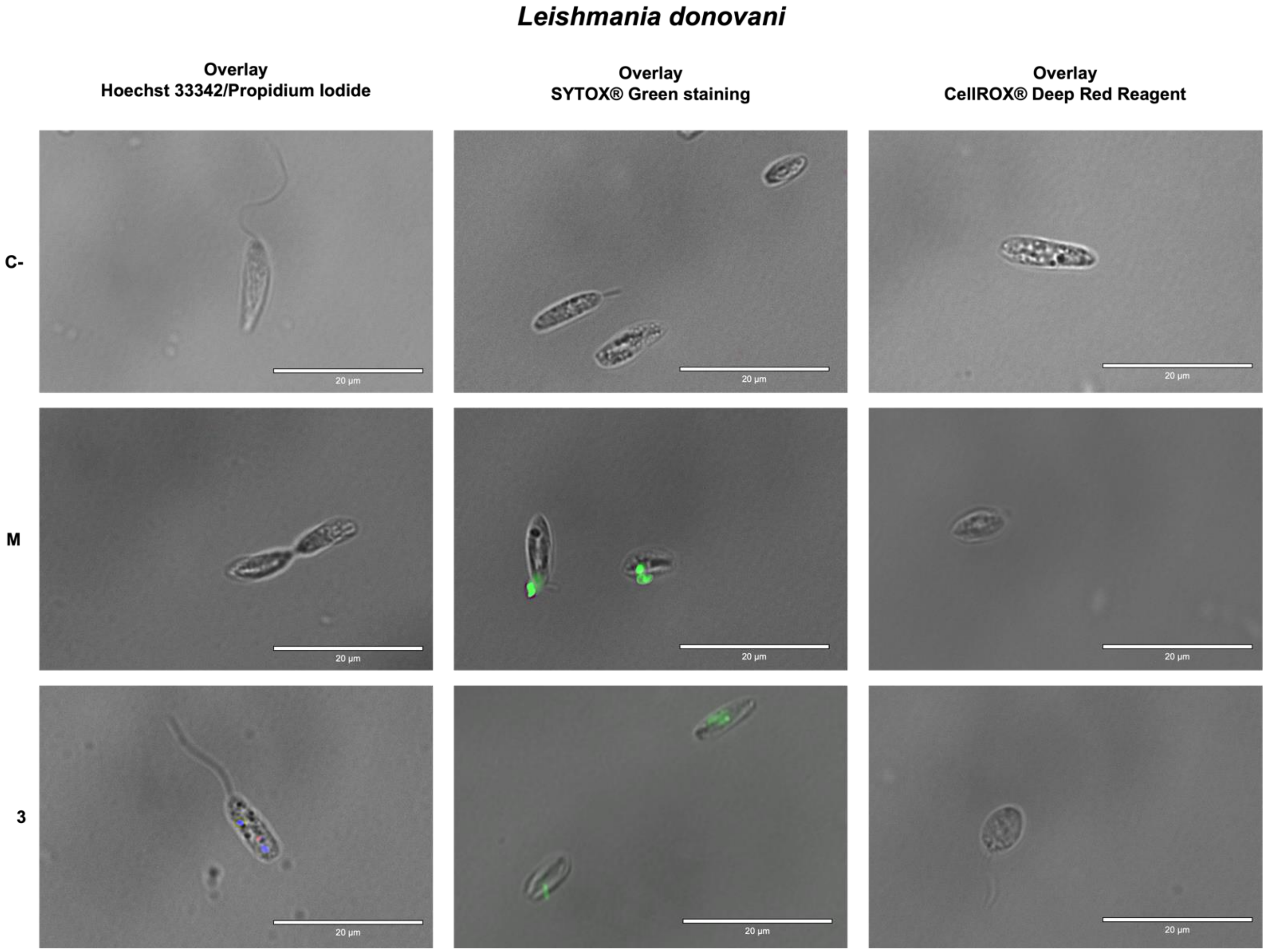

Figure S3. Overlays of the detection of chromatin condensation by Vybrant® Apoptosis Assay Kit n°5, Hoechst 33342/Propidium Iodide, changes on the plasmatic membrane permeability by SYTOX® Green staining, and reactive oxygen species by CellROX® Deep Red Reagent in *T. cruzi*.

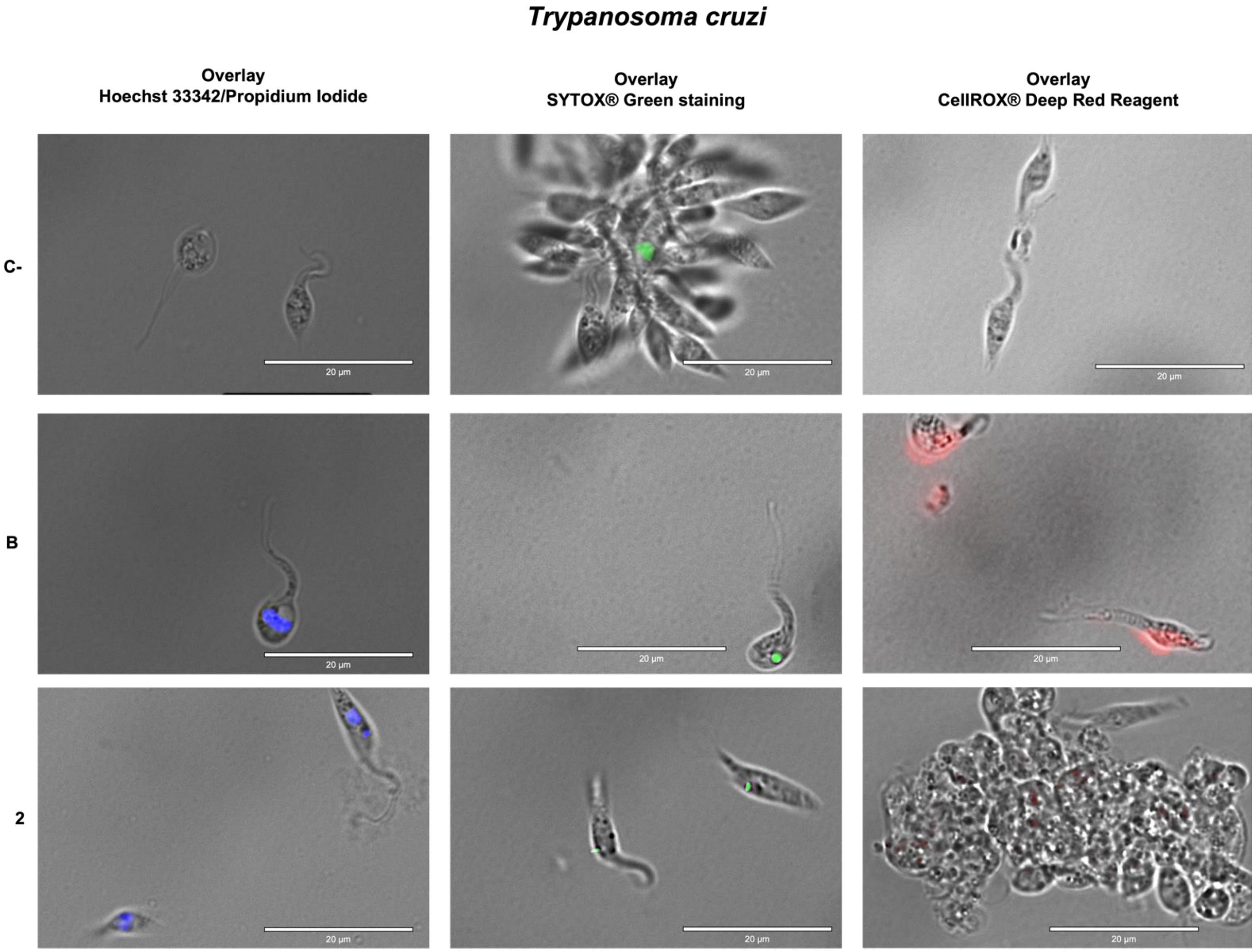

Table S1. Physical and spectroscopic data of compounds **1** and **2** [NMR: $CDCl_3$, 500 MHz, 298K]

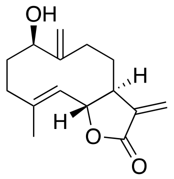

Artemorin (**1**)

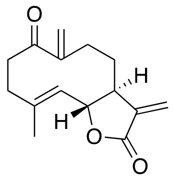

Anhydroartemorin (**2**)

nº C	δ ^{13}C	δ ^{1}H	m	*J* (Hz)	δ ^{13}C	δ ^{1}H	m	*J* (Hz)
**1**	78.3	3.97	m	-	204.9	-	-	-
**2**	32.2	1.95 – 1.98	m	-	35.3	2.54	m	-
		2.15 – 2.20	m			3.16	m	-
**3**	36.1	1.95 – 1.98	m	-	38.3	2.36	ddd	2.9, 8.7, 9.3
		2.15 – 2.20	m			2.54	m	-
**4**	141.1	-	-	-	143.0	-	-	-
**5**	122.8	5.22	d	14.2	125.3	5.09	d	10.0
**6**	80.3	4.40	m	-	81.2	4.33	dd	9.7, 10.0
**7**	43.3	2.81	m	-	49.9	2.54	m	-
**8**	25.6	2.30 (2H)	m	-	28.1	2.54 (2H)	m	-
**9**	31.1	1.62	m		29.1	1.42	m	-
		2.30	m	-		2.24	m	-
**10**	157.9	-	-	-	142.4	-	-	-
**11**	145.9	-	-	-	136.6	-	-	-
**12**	165.0	-	-	-	169.5	-	-	-
**13**	118.1	5.45	d	3.5	119.2	5.48	d	3.5
		6.17	d	3.2		6.22	d	3.5
**14**	110.7	4.87	s	-	123.8	5.66	s	-
		5.21	s	-		5.82	s	-
**15**	17.8	1.72	s	-	17.1	1.76	s	-

$[\alpha]^{25}_{D}$ +70.0 (*c* 0.08, $CHCl_3$)
IR ν_{max}: 3340, 2918, 2849, 1758, 1667 and 1446 cm^{-1}
ESI-HRMS: $C_{15}H_{20}O_3Na$ $[M+Na]^+$; *m/z* 271.1307 (Calcd. 271.1310)

$[\alpha]^{25}_{D}$ +60.0 (*c* 0.02, $CHCl_3$)
IR ν_{max}: 2924, 2852, 1765,1712, 1668 and 1454 cm^{-1}
ESI-HRMS: $C_{15}H_{18}O_4Na$ $[M+Na]^+$; *m/z* 269.1198 (Calcd. 269.1154)

Table *S2*. Physical and spectroscopic data of compounds **3-5** [NMR: $CDCl_3$, 500 MHz, 298K]

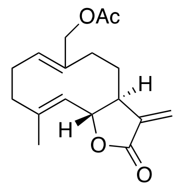

cis,trans-Costunolide-14-acetate (**3**)

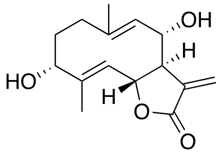

Tatridin A (**4**)

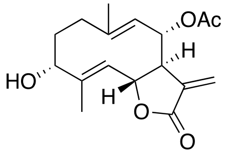

Tatridin A acetate (**5**)

nº C	δ ^{13}C	δ ^{1}H	m	*J* (Hz)	δ ^{13}C	δ ^{1}H	m	*J* (Hz)	δ ^{13}C	δ ^{1}H	m	*J* (Hz)
**1**	97.6	6.00	d	9.2	35.2	(α) 2.30	dd	6.1, 11.6	35.3	(α) 2.30	dd	6.7, 12.3
						(β) 1.93	dd	6.1, 11.6		(β) 1.91	m	-
**2**	31.2	1.79	m	-	27.2	(α) 1.74	m	-	27.3	(α) 1.76	dd	6.7, 12.1
		2.15	m	-		(β) 2.00	dd	6.2, 12.0		(β) 2.03	dd	6.7, 12.1
**3**	37.2	2.31 (2H)	m	-	66.7	4.39	m	-	66.9	4.40	m	-
**4**	140.7	-	-	-	142.2	-	-	-	142.9	-	-	-
**5**	123.2	5.18	d	9.5	126.8	5.33	d	10.2	126.4	5.33	d	9.7
**6**	79.3	4.66	dd	9.5, 9.5	74.0	4.54	dd	9.4, 10.2	74.1	4.62	dd	9.4, 9.7
**7**	43.9	2.76	dddd	3.2, 5.8, 5.9, 9.5	52.3	2.80	ddd	3.1, 9.4, 10.3	49.2	3.03	dd	9.4, 9.9
**8**	25.4	1.69	m	-	71.1	4.47	dd	3.4, 10.3	73.1	5.39	dd	9.9, 10.6
		1.95	m	-								
**9**	31.6	2.14	m	-	129.9	4.99	d	10.3	126.0	4.87	d	10.6
		2.31	m	-								
**10**	157.9	-	-	-	135.2	-	-	-	137.9	-	-	-
**11**	135.3	-	-	-	135.9	-	-	-	136.9	-	-	-
**12**	170.1	-	-	-	169.3	-	-	-	169.1	-	-	-
**13**	118.9	5.46	d	3.2	123.6	6.21	d	3.1	122.9	5.75	d	3.3
		6.19	d	3.2		6.31	d	3.1		6.29	d	3.3
**14**	87.1	4.09	d	3.0	15.6	1.78	s	-	15.6	1.90	s	-
		4.20	d	3.0								
**15**	16.6	1.83	s	-	16.7	1.83	s	-	16.7	1.83	s	-
	169.6 (C=O)	-	-	-	-	1.47 (OH)	d	3.0	169.8 (C=O)	-	-	-
	21.1 (Me-O-)	2.09	s	-	-	1.65 (OH)	d	3.4	21.0 (Me-O-)	2.09	s	-

[α]$^{25}_{D}$ +38.3 (*c* 0.06, $CHCl_3$)
IR ν_{max}: 2925, 2852, 2360, 1766, 1667 and 1445 cm^{-1}
ESI-HRMS: $C_{17}H_{22}O_5Na$ $[M+Na]^+$; *m/z* 329.1357 (Calcd. 329.1365)

[α]$^{25}_{D}$ -33.3 (*c* 0.03, $CHCl_3$)
IR ν_{max}: 3363, 2918, 2849, 2360, 1759 and 1666 cm^{-1}
ESI-HRMS: $C_{15}H_{20}O_4Na$ $[M+Na]^+$; *m/z* 287.1259 (Calcd. 287.1259)

[α]$^{25}_{D}$ -+26.7 (*c* 0.06, $CHCl_3$)
IR ν_{max}: 3374, 2918, 2850, 1771, 1667and 1438 cm^{-1}
ESI-HRMS: $C_{17}H_{22}O_5Na$ $[M+Na]^+$; *m/z* 329.1368 (Calcd. 329.1365)

Table S3. Physical and spectroscopic data of compounds **6** and **7** [NMR: $CDCl_3$, 500 MHz, 298K]

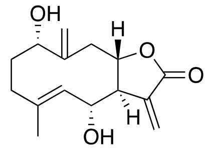

Tanachin (**6**)

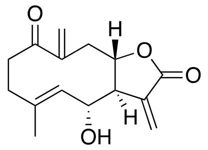

Tamirin (**7**)

nº C	δ ^{13}C	δ ^{1}H	m	*J* (Hz)	δ ^{13}C	δ ^{1}H	m	*J* (Hz)
	**Tanachin (6)**				**Tamirin (7)**			
**1**	70.4	3.84	m	-	203.0	-	-	-
**2**	31.2	(α) 2.17	m	-	36.5	2.55	m	-
		(β) 2.05	ddd	3.8, 8.8, 8.8		3.28	ddd	5.4, 5.4, 8.6
**3**	34.4	(α) 2.11	m	-	35.9	2.41	ddd	5.4, 5.6, 8.6
		(β) 2.26	ddd	3.3, 5.5, 8.8		2.55	m	-
**4**	136.0	-	-	-	136.8	-	-	-
**5**	131.6	5.05	d	10.0	131.9	5.08	d	10.3
**6**	71.5	4.28	ddd	3.3, 10.0, 10.0	70.1	4.16	ddd	3.0, 10.3, 10.3
**7**	51.9	2.83	ddd	3.0, 6.5, 10.0	50.2	2.74	ddd	2.0, 9.7, 10.3
**8**	79.0	3.97	m	-	76.5	3.96	m	-
**9**	42.0	(α) 2.39	dd	10.3, 14.0	40.1	(α) 2.15	dd	11.7, 11,7
		(β) 2.96	d	14.0		(β) 3.42	d	11,7
**10**	146.7	-	-	-	146.3	-	-	-
**11**	137.1	-	-	-	135.6	-	-	-
**12**	170.0	-	-	-	169.6	-	-	-
**13**	125.7	6.20	d	3.0	126.2	6.17	d	2.0
		6.37	d	3.0		6.38	d	2.0
**14**	115.2	5.12	sa	-	125.2	5.79	d	1.8
		5.17	sa	-		5.83	sa	-
**15**	17.7	1.71	s	-	17.3	1.66	s	-
	-	1.73 (OH)	d	3.3				

Tanachin (6):
[α]$^{25}_{D}$ +0.8 (*c* 0.07, $CHCl_3$)
IR ν_{max}: 3390, 2925, 2855, 1756, 1659 and 1446 cm^{-1}
ESI-HRMS: $C_{15}H_{20}O_4Na$ $[M+Na]^+$; *m/z* 287.1253 (Calcd. 287.1259)

Tamirin (7):
[α]$^{25}_{D}$ -23.3 (*c* 0.06, $CHCl_3$)
IR ν_{max}: 3444, 2922, 2851, 1758, 1672 and 1443cm^{-1}
ESI-HRMS: $C_{15}H_{18}O_4Na$ $[M+Na]^+$; *m/z* 285.1104 (Calcd. 285.1103)

Table S4. Physical and spectroscopic data of compounds **8** and **9** [NMR: $CDCl_3$, 500 MHz, 298K]

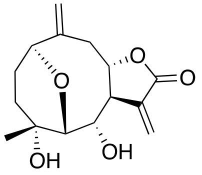

Isobadgerin (**8**)

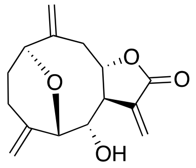

Dehydroxyisobadgerin (**9**)

nº C	$\delta^{13}C$	$\delta^{1}H$	m	*J* (Hz)	$\delta^{13}C$	$\delta^{1}H$	m	*J* (Hz)
**1**	77.8	4.44	m	-	75.4	4.46	dd	5.1, 5.7
**2**	24.4	(α) 1.67	m	-	26.9	(α) 1.78	dddd	2.6, 5.0, 5.3, 15.7
		(β) 2.05	m	-		(β) 2.26	dddd	2.6, 5.1, 5.7, 15.7
**3**	38.0	(α) 1.67	m	-	28.1	(α) 2.46	m	
		(β) 2.05	m	-		(β) 2.35	ddd	5.0, 5.3, 15.7
**4**	70.8	-	-	-	144.2			
**5**	77.8	2.86	sa	2.2	73.0	3.49	sa	-
**6**	72.3	4.22	dd	8.4, 10.3	72.3	4.20	ddd	2.3, 6.8, 7.9
**7**	54.8	3.21	ddd	3.5, 9.5, 10.3	53.6	3.32	ddd	3.5, 7.9, 10.4
**8**	81.8	4.10	dd	9.5, 12.4	81.6	4.06	dd	10.4, 10.5
**9**	42.0	(α) 2.45	dd	10.75, 12.46	42.8	(α) 2.96	d	12.7
		(β) 2.95	d	12.45		(β) 2.62	dd	10.5, 12.7
**10**	145.1	-	-	-	149.9	-	-	-
**11**	127.8	-	-	-	139.2	-	-	-
**12**	169.0	-	-	-	169.2	-	-	-
**13**	121.0	6.02	d	3.14	121.4	6.07	d	3.5
		6.21	d	3.55		6.24	d	3.5
**14**	117.5	5.12	d	1.70	117.6	5.13	d	2.0
		5.35	sa	-		5.38	sa	-
**15**	21.7	1.52	s	-	107.1	4.95	sa	-
						4.98	d	1.9
					-	2.03 (OH)	d	6.8

[α]$^{25}_{D}$ -12.2 (*c* 0.09, $CHCl_3$)
IR ν_{max}: 3362, 2921, 2851, 1766, 1660 and 1455 cm^{-1}
ESI-HRMS: $C_{15}H_{20}O_5Na$ $[M+Na]^+$; *m/z* 303.1209 (Calcd. 303.1208)

[α]$^{25}_{D}$ +60.0 (*c* 0.12, $CHCl_3$)
IR ν_{max}: 3457, 2918, 2850, 1762, 1661 and 1436 cm^{-1}
ESI-HRMS: $C_{15}H_{18}O_4Na$ $[M+Na]^+$; *m/z* 285.1103 (Calcd. 285.1103)

Table S5. Physical and spectroscopic data of (-)-nephtediol (**10**) [NMR: $CDCl_3$, 500 MHz, 298K]

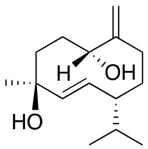

(-) Nephthediol (**10**)

C nº	δ ^{13}C	δ ^{1}H	mult.	*J* (Hz)
**1**	78.3	3.94	d	8.7
**2**	28.3	1.62	m	
		1.93	m	
**3**	38.5	1.43	m	
		1.84	m	
**4**	72.3			
**5**	137.4	5.22	d	15.18
**6**	130.0	5.30	dd	9.5, 15.25
**7**	49.7	1.87	m	
**8**	29.5	1.55	m	
		1.96	m	
**9**	28.2	1.82	m	
		2.25	dd	12.0, 12.8
**10**	151.1			
**11**	32.3	1.50	dd	6.7, 13.5
**12**	20.5	0.84	d	6.7
**13**	20.6	0.89	d	6.7
**14**	111.4	4.90	s	
		5.13	s	
**15**	23.9	1.27	s	

$[\alpha]^{25}_{D}$ -70.0 (*c* 0.02, $CHCl_3$)

IR ν_{max}: 3314, 2927, 2869, 1723, 1670 and 1454 cm^{-1}

ESI-HRMS: $C_{15}H_{26}O_2Na$; $[M+Na]^+$ *m/z* 261.1833 (Calcd. 261.1831)

Table S6. Physical and spectroscopic data of compounds **11-13** [NMR: $CDCl_3$, 500 MHz, 29

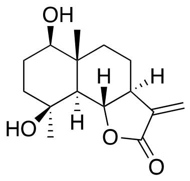

4β−Hydroxyarbusculin A (**11**)

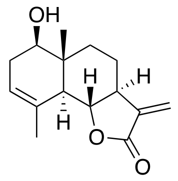

Santamarine (**12**)

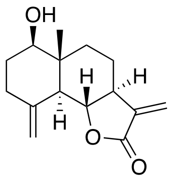

Reynosin (**13**)

nº C	δ ^{13}C	δ ^{1}H	m	*J* (Hz)	δ ^{13}C	δ ^{1}H	m	*J* (Hz)	δ ^{13}C	δ ^{1}H	m	*J* (Hz)
**1**	78.3	3.46	m	-	75.0	3.68	ddd	5.0, 6.0, 10.0	78.1	3.53	dd	4.5, 11.5
**2**	28.5	(α) 1.64	m	-	32.8	(α) 1.97	m	-	31.2	(α) 1.85	m	-
		(β) 1.76	dd	5.3, 12.0		(β) 2.40	m	-		(β) 1.57	m	-
**3**	38.0	(α) 1.64	m	-	21.2	5.35	d	2.7	33.5	(α) 2.14	m	-
		(β) 1.81	dd	3.0, 12.0						(β) 2.34	ddd	1.0, 5.1, 13.8
**4**	71.9	-	-	-	133.1	-	-	-	143.3	-	-	-
**5**	57.1	1.86	d	11.5	51.2	2.35	d	11.0	52.9	2.19	d	10.9
**6**	80.8	4.12	dd	11.5, 11.5	81.2	3.95	dd	11.0, 11.0	79.6	4.03	dd	10.9, 10.9
**7**	50.7	2.60	dddd	3.1, 3.3, 11.5, 11.5	51.0	2-50	ddd	3.2, 11.0, 12.8	49.4	2.54	ddd	3.1, 10.9, 11.2
**8**	22.0	(α) 2.02	ddd	3.3, 11.5, 13.0	21.2	(α) 2.10	dddd	3.9, 3.9, 6.4, 12.9	21.4	(α) 2.09	m	-
		(β) 1.57	m	-		(β) 1.67	dddd	3.4, 12.8, 12.8, 12.9		(β) 1.60	m	-
**9**	39.1	(α) 1.30	m	-	34.4	(α) 1.31	ddd	3.9, 12.9, 12.9	35.5	(α) 1.36	ddd	3.9, 13.1, 13.1
		(β) 2.01	ddd	3.3, 3.3., 13.0		(β) 2.06	ddd	4.0, 6.4, 12,9		(β) 2.10	m	-
**10**	42.1	-	-	-	40.5	-	-	-	42.9	-	-	-
**11**	138.9	-	-	-	138.5	-	-	-	139.1	-	-	-
**12**	171.3	-	-	-	170.6	-	-	-	170.5	-	-	-
**13**	118.0	5.46	d	3.1	116.7	5.41	d	3.2	117.0	5.42	d	3.1
		6.13	d	3.1		6.08	d	3.2		6.09	d	3.1
**14**	13.9	0.98	s	-	11.0	0.88	s	-	11.6	0.82	s	-
**15**	24.1	1.37	s	-	23.2	1.84	s	-	110.1	4.87	d	1.0
		3.09 (OH)	s	-		1.41 (OH)	d	5.0		4.99	sa	-

[α]^{25}$_{D}$ +15.0 (*c* 0.10, $CHCl_3$)
IR ν_{max}: 3418, 2931, 2871, 1769, 1460 and 1411 cm^{-1}
ESI-HRMS: $C_{15}H_{22}O_4Na$; $[M+Na]^+$ *m/z* 289.1417 (Calcd. 289.1416)

[α]^{25}$_{D}$ +68.6 (*c* 0.07, $CHCl_3$)
IR ν_{max}: 3347, 2918, 2848, 1762, 1439 and 1412 cm^{-1}
ESI-HRMS: $C_{15}H_{20}O_3Na$; $[M+Na]^+$ *m/z* 271.1315 (Calcd. 271.1310)

[α]^{25}$_{D}$ +82.0 (*c* 0.20, $CHCl_3$)
IR ν_{max}: 3454, 2928, 2851, 1768, 1460 and 1413 cm^{-1}
ESI-HRMS: $C_{15}H_{20}O_3Na$; $[M+Na]^+$ *m/z* 271.1308 (calc. 271.1310)
